# Antimalarial drugs for preventing malaria during pregnancy and the risk of low birth weight: a systematic review and meta-analysis of randomized and quasi-randomized trials

**DOI:** 10.1186/s12916-015-0429-x

**Published:** 2015-08-14

**Authors:** Flory Tsobo Muanda, Sonia Chaabane, Takoua Boukhris, Fabiano Santos, Odile Sheehy, Sylvie Perreault, Lucie Blais, Anick Bérard

**Affiliations:** Faculty of Pharmacy, University of Montreal, 2940 Chemin de Polytechnique, Montreal, QC H3T 1J4 Canada; Research Center, CHU Sainte-Justine, 3175 Chemin de la Côte-Sainte-Catherine, Montreal, QC H3T 1C5 Canada; Faculty of Medicine, McGill University, 3605 Rue de la Montagne, Montreal, QC H3G 2M1 Canada

## Abstract

**Background:**

It is known that antimalarial drugs reduce the risk of low birth weight (LBW) in pregnant patients. However, a previous Cochrane review did not evaluate whether the level of antimalarial drug resistance could modify the protective effect of antimalarial drugs in this regard. In addition, no systematic review exists comparing current recommendations for malaria prevention during pregnancy to alternative regimens in Africa. Therefore, we conducted a comprehensive systematic review and meta-analysis to assess the efficacy of antimalarial drugs for malaria prevention during pregnancy in reducing the risk of LBW.

**Methods:**

We searched PubMed, Embase and the Cochrane Central Register of Controlled Trials (CENTRAL) for articles published up to 21 November 2014, in English or French, and identified additional studies from reference lists. We included randomized and quasi-randomized studies reporting LBW as one of the outcomes. We extracted data and assessed the risk of bias in selected studies. All pooled analyses were based on a random effect model, and we used a funnel plot and trim and fill method to test and adjust for publication bias.

**Results:**

A total of 25 studies met the inclusion criteria (37,981 subjects). Compared to no use, all combined antimalarial drugs were associated with a 27 % (RR 0.73, 95 % CI 0.56–0.97, ten studies) reduction in the risk of LBW. The level of antimalarial drug resistance modified the protective effect of the antimalarial drug used for prevention of LBW during pregnancy. Sulfadoxine-pyrimethamine was not associated with a reduction in the risk of LBW in regions where the prevalence of the dihydropteroate synthase 540E mutation exceeds 50 % (RR 0.99, 95 % CI 0.80–1.22, three studies). The risk of LBW was similar when sulfadoxine-pyrimethamine was compared to mefloquine (RR 1.05, 95 % CI 0.86–1.29, two studies).

**Conclusion:**

Prophylactic antimalarial drugs and specifically sulfadoxine-pyrimethamine may no longer protect against the risk of LBW in areas of high-level resistance. In Africa, there are currently no suitable alternative drugs to replace sulfadoxine-pyrimethamine for malaria prevention during pregnancy.

**Electronic supplementary material:**

The online version of this article (doi:10.1186/s12916-015-0429-x) contains supplementary material, which is available to authorized users.

## Background

Low birth weight (LBW) is defined as a birth weight of a live born infant of less than 2,500 g (5.5 lb) regardless of gestational age [1]. LBW is one of the leading causes of neonatal and infant mortality in the world and may be the result of a short gestational period, intrauterine growth restriction (IUGR), or a combination of both events [1]. Infants born with this adverse outcome are at higher risk of early growth retardation, infectious diseases, developmental delay, death during infancy and chronic disease in adulthood [2, 3]. It is estimated that over 4 million children are born each year with LBW in Africa, where malaria infection accounts for up to 560,000 (14 %) LBW infants and 11 % of LBW-related infant mortality [4]. Malaria is a mosquito-borne infectious disease transmitted by a parasitic protozoan in the *Plasmodium* genus**.** This disease leads to maternal anemia and placental parasitemia, which are both known risk factors for LBW. This may explain the high LBW prevalence observed in regions where malaria is endemic, and hence making the disease a potentially modifiable necessary cause of LBW. Therefore, the use of antimalarial drugs appears to be one of the most appropriate interventions to prevent the deleterious effect of malaria during pregnancy and to reduce child mortality as recommended by the Millennium Development Goals (MDGs) [5]. A recent Cochrane review published in 2014 reported that compared to placebo, prophylactic antimalarial drug use during pregnancy reduced the risk of LBW by 27 % among women in their first or second pregnancy [6]. Previously, another meta-analysis showed that the use of three or more doses of sulfadoxine-pyrimethamine compared to two doses was associated with a reduction of the risk of LBW by 23 % among HIV-negative women [7].

World Health Organization (WHO) guidelines for the treatment of malaria recommend that first-line treatment should be changed if the total failure rate exceeds 10 % [8]. Current evidence suggests that the efficacy of antimalarial drugs in preventing LBW may decrease with *Plasmodium* resistance [9], but the threshold at which these drugs will fail to reduce the risk of LBW remains unclear. An earlier meta-analysis published in 2007 showed that the level of drug resistance had no effect on the efficacy of intermittent preventive treatment (IPT) during pregnancy with sulfadoxine-pyrimethamine in reducing the risk of LBW. However, the range of resistance (19–26 %) identified in those studies was narrow, which limited the generalizability of this finding [10]. In contrast, a cohort study conducted in central Africa in 2012 showed that the level of drug resistance may modify the effectiveness of sulfadoxine-pyrimethamine. However, this result should be interpreted with caution given the study design limitations [11]. In addition, it is currently known that high prevalence of quintuple mutation (dihydropteroate synthase 540 E mutation, dhps 540E) and sextuple mutation (additional dhps 581G or dihydrofolate reductase, dhfr 164 L mutation to quintuple mutation) in *Plasmodium falciparum* infections may interfere with sulfadoxine-pyrimethamine effectiveness [12–15]. The 2014 Cochrane review did not evaluate drug resistance as a modifier of the protective effect of antimalarial drugs in reducing the risk of LBW. In addition, there is a lack of head-to-head randomized controlled trials (RCTs) that evaluate potential alternatives to IPT during pregnancy with sulfadoxine-pyrimethamine. However, given the increasing resistance to this drug, some head-to-head RCTs comparing sulfadoxine-pyrimethamine to mefloquine or antibiotics have been published recently [16–18], but no updated synthesis including these new trials are available in the literature.

Therefore, the objectives of our systematic review were: 1) to quantify the protective effect of antimalarial drugs used for the prevention of malaria during pregnancy in reducing the risk of LBW compared to no use of antimalarial drugs; 2) to examine whether the level of drug resistance and gravidity could modify the protective effect of antimalarial drugs in reducing the risk of LBW; 3) to evaluate the risk of LBW associated with the use of three doses or more of the main types of antimalarial drugs compared to the use of two doses of these drugs; and 4) to compare the risk of LBW between women taking sulfadoxine-pyrimethamine and those taking different antimalarial drug alternatives for malaria prevention during pregnancy.

## Methods

### Data sources and search strategy

We followed the Preferred Reporting Items for Systematic Reviews and Meta-Analysis (PRISMA) [19]. For a complete PRISMA checklist, see Additional file 1. We systematically searched PubMed, Embase and the Cochrane Central Register of Controlled Trials (CENTRAL) for all relevant articles, reporting associations between LBW and gestational exposure to antimalarial drugs used for the prevention of malaria during pregnancy up to 21 November 2014, with the following key words: ‘malaria’; and ‘low birth weight’. Details of the search strategy for PubMed are available and described in Additional file 2. The search strategies for other databases are available upon request. The reference lists of retrieved articles were reviewed in order to identify studies that were not obtained from the preliminary literature.

### Study selection

Eligibility criteria for study selection included: 1) RCTs and quasi-randomized controlled trials containing data of pregnant women exposed to any type of antimalarial drug used for malaria prevention, as well as a control group (no use of antimalarial drug, placebo, or other type of antimalarial), and with data reporting the outcome of interest (LBW). RCTs were defined as clinical trials in which individuals or other units were assigned to different treatment groups using randomization allocation, such as random number, computer-generated random sequences, coin tossing and drawing lots. Quasi-randomized studies are clinical trials in which individuals or other units are assigned to different treatment groups using non-strictly random methods of allocation. Examples of quasi-random methods of assignment include alternation, date of birth and medical record number [20]; 2) studies published in French or English; and 3) studies that reported a *P. falciparum* infection. We excluded observational studies (cohort, case control and cross-sectional studies), case reports, case series and unpublished data (grey literature). Studies conducted among HIV-infected women were also discarded. Indeed, a recent meta-analysis that included HIV-positive pregnant women was available in the literature [7] and since then only one head-to-head RCT conducted among HIV-positive pregnant women has been published [21].

**Fig. 1:**
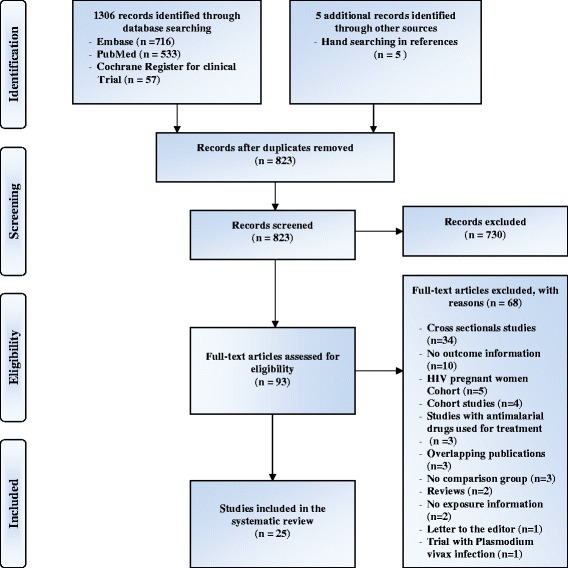
PRISMA flow chart for systematic review of antimalarial drugs for malaria prevention during pregnancy and the risk of low birth weight (LBW)

After the initial search strategy, two independent reviewers (FTM and AB) used the inclusion and exclusion criteria to screen titles of abstracts and assessed only full texts of relevant studies for eligibility. Disagreements were resolved by consensus. All studies that did not meet inclusion criteria were excluded with reasons provided in a flow chart (Fig. 1).

### Data extraction

Two investigators (FTM and AB) extracted the following information in a standardized form: first author’s name; publication year; period of study; location; study design; number of women enrolled; risk of LBW in exposed and control group; dropout rates in each trial; local malaria transmission rates; and level of antimalarial drug resistance (clinical and molecular resistance).

Clinical resistance to antimalarial drugs was defined by treatment failure rates at day 14 or 28 in symptomatic children aged 6–59 months in the study region [22]. If available, data on transmission level and therapeutic failure to antimalarial drugs in children aged 6–59 months in the study region were collected from each RCT included in our review.

We used this subpopulation to evaluate efficacy of antimalarial drugs, given that most RCTs included in our review were carried out when assessment of efficacy of these drugs involved children under 5 years old of age. Moreover, a previous meta-analysis also used a therapeutic failure rate of children under 5 years old to assess the effect of sulfadoxine-pyrimethamine resistance on the efficacy of intermittent preventive therapy for malaria control during pregnancy [10].

Molecular resistance to sulfadoxine-pyrimethamine was defined by the prevalence of molecular markers of *P. falciparum* resistance to sulfadoxine-pyrimethamine (dhps 540E) in symptomatic children aged 6–59 months. We used data obtained from a database that mapped dhfr and dhps gene distribution associated with sulfadoxine-pyrimethamine resistance in Africa [9, 12, 23]. This map is currently used as a tool to track the spatial extent and temporal patterns of sulfadoxine-pyrimethamine resistance mutations across the African continent [12].

This method is the gold standard for monitoring antimalarial drug efficacy and helps policy makers shape recommendations for malaria treatment and prophylaxis [24].

Risk ratios (RRs; intention-to-treat and per-protocol estimates) along with 95 % confidence intervals (CIs) were extracted from all included studies. When the measure of association was not reported, we calculated unadjusted risk ratios by means of the Mantel–Haenszel chi-squared test. Therefore, we used the risk ratio as the main effect measure in this review.

### Risk of bias in individual studies

The risk of bias for each study included in the review was assessed independently by two authors using the Cochrane Collaboration’s tool for assessing risk of bias of RCTs [25]. Disagreement was resolved through consensus after discussion in integrative session. The tool covers six bias domains: 1) sequence generation describes the method used to generate the allocation sequence in sufficient detail to allow an assessment of whether it should produce comparable groups; 2) allocation concealment describes the method used to conceal the allocation sequence in sufficient detail to determine whether intervention allocations could have been foreseen before or during enrolment; 3) blinding of participants and personnel describes all measures used, if any, to blind trial participants and researchers from knowledge of which intervention a participant received; 4) incomplete outcome data describes the completeness of outcome data for each main outcome including attrition and exclusions from the analysis; 5) selective reporting describes how selective outcome reporting was examined and what was found; and 6) other source of bias describes any important concerns about bias not covered in the other domains in the tool [25]. Based on empirical and theoretical considerations [25], RCTs with inadequate random sequence generation, allocation concealment, attrition [26] or reporting bias were considered as studies with a high risk of bias. When sufficient information was not provided on these different domains of bias to allow a definite judgement, we considered that the risk of bias was unclear. In contrast, when a study was free of these biases, we considered the risk of bias as low.

### Statistical analysis

We calculated the summary risk ratios and 95 % confidence intervals using a random effect model (method of DerSimonian and Laird) due to differences between study populations and interventions [27]. This model considers the variation within and between RCTs in their calculation of the overall effect size [20]. Given that the RCTs included in our review were carried out across different countries in Africa, with different populations, interventions and levels of antimalarial drug resistance [9, 12, 23], we assumed that the random effect model was the most appropriate model to calculate the summary estimate for all analyses.

In the sensitivity analysis, we used a fixed effect model to test the robustness of our findings [20]. Pooled estimates of each meta-analysis and estimates of individual studies also with their 95 % CI were presented in a forest plot. Heterogeneity was explored graphically and quantified with the I^2^ statistic, which provides the percentage of the variability in effect estimates that is due to heterogeneity rather than sampling error (chance) [28]. When the value of the I^2^ statistic was found to be higher than 50 %, representing significant heterogeneity [20], we identified outliers using a Galbraith plot. We attempted to explain this heterogeneity by stratification according to the level of clinical resistance to antimalarial drugs (treatment failure rates in symptomatic children at day 14 or 28), gravidity and risk of bias of studies included.

To investigate the association between clinical resistance and the efficacy of antimalarial drugs in reducing the risk of LBW, we used a 10 % threshold for treatment failure rate, which is the current cut-off recommended by the WHO for policy change in malaria areas [8]. We also restricted our analysis of trials conducted in East Africa, where the prevalence of the dihydropteroate synthase 540E mutation (a proxy of high-level sulfadoxine-pyrimethamine resistance) exceeds 50 %, to examine the association between molecular resistance to sulfadoxine-pyrimethamine and its efficacy in reducing LBW [23]. This threshold for molecular markers has also been used, as recommended by the WHO, stating that intermittent preventive treatment of infants (IPTi) with sulfadoxine-pyrimethamine should not be implemented when the prevalence of the dhps 540E mutation among infected individuals exceeds 50 % [23].

We evaluated publication bias by visual inspection of a funnel plot (eyeball test) and by Egger’s [29] and Begg’s test [30], and adjusted for possible publication bias by means of the trim and fill method [31]. We performed a sensitivity analysis with a fixed effect model (inverse variance method) and conducted additional analysis by removing outliers identified by a Galbraith plot.

We also investigated the influence of each individual study on the overall meta-analysis summary estimate after removal of each trial one at a time from the meta-analysis. We quantified the difference between risk estimate of trials with low risk of bias compared to trials with high risk and unclear risk of bias using random effect meta-regression in our main meta-analysis. Pooled analysis was based on intention-to-treat estimates when provided in the trial. Otherwise, per-protocol estimates were used. All analyses were performed using Stata version 12.1 (StataCorp LP, College Station, TX, USA).

## Results

### Study selection

A total of 1,306 articles were initially identified. After removing duplicates, 818 articles remained for further consideration. Screening article titles and abstracts led to the exclusion of an additional 730 articles, and the assessment of 88 full-text articles for possible inclusion. After reviewing the reference list of eligible articles, five additional articles were added for possible inclusion. Of those 93 articles, 25 met the inclusion criteria [16–18, 32–53] and 68 were excluded. The list of the reasons for the exclusions is provided (Fig. 1). The flow of studies through the screening process of the review is shown in Fig. 1 and reported based on PRISMA guidelines.

### Study characteristics

Data from 37,981 pregnant women across the included studies (range: 266 to 5,775) were analyzed in our review. Ten studies compared any type of antimalarial drug (chloroquine, sulfadoxine-pyrimethamine, mefloquine, dapsone-pyrimethamine) to a placebo or no use of antimalarial drug; five studies compared three doses or more versus two doses of sulfadoxine-pyrimethamine; two studies compared weekly doses versus two doses of chloroquine; five studies compared IPT with sulfadoxine-pyrimethamine versus weekly chloroquine; two studies compared IPT with sulfadoxine-pyrimethamine versus IPT with mefloquine; and the remaining studies compared IPT with amodiaquine-sulfadoxine-pyrimethamine compared to IPT with sulfadoxine-pyrimethamine alone (one study), and IPT with sulfadoxine-pyrimethamine versus daily cotrimoxazole (one study).

The median dropout rate of studies included in the review was 14.1 % (interquartile range (IQR): 8.55–21.45 %; range: 1.5–51 %). The rate of loss to follow-up did not significantly differ between the exposed and control group when reasons were provided in the study. Four trials showed a significant difference in the rate of adverse events between the exposed and control group [16, 39, 40, 46]. The first trial conducted by Clerk and colleagues showed that women taking amodiaquine-sulfadoxine-pyrimethamine were more likely to report an adverse event than those taking sulfadoxine-pyrimethamine alone (RR 1.88, 95 % CI 1.70–2.07), but no women were withdrawn from the study because of an adverse event [39]. The second trial carried out in Thailand by Nosten and colleagues reported a significant difference of the rate of stillbirth between women who were taking mefloquine compared to women exposed to a placebo (*P* <0.01) in the first phase of the study, but as this increase was unexpected the author conducted a second phase in the trial and pooled the data from the two periods that showed no significant difference in the rate of stillbirth between women exposed to mefloquine and control (*P* = 0.13) [46]. The third trial, which was a multicentre study, showed that the number of women with serious adverse events was higher in the mefloquine group compared to the sulfadoxine-pyrimethamine group, but a causal relationship could not be established by members from the data and safety monitoring board (DSMB) of the trial [16]. The fourth trial conducted by Briand and colleagues in Benin showed that adverse events were more commonly associated with the use of mefloquine compared to sulfadoxine-pyrimethamine (prevalence: 78 % versus 32 %; *P* <0.01) [40].

No significant differences in the rate of adverse events or foetal loss were found in the remaining studies [17, 18, 32–38, 41–45, 47–53]. Ten trials (40 %) reported using intention-to-treat analysis [17, 18, 32, 33, 41, 43, 44, 47, 49, 53]; two trials (8 %) carried out a modified intention-to-treat analysis [16, 40]; and the remaining 13 studies (52 %) used a per-protocol analysis [34–39, 42, 45, 46, 48, 50–52]. A summary of included studies evaluating the use of antimalarial drugs for preventing malaria during pregnancy and the risk of LBW is shown in Additional file 3: Table S1.

### Risk of bias in individual studies

Of the 25 studies included, only four studies (16 %) were considered as having a low risk of bias [16, 37, 49, 53] and 17 studies (68 %) were judged as presenting a high risk of bias [17, 18, 32–36, 38, 43–48, 50–52]. The risk of bias was unclear in the remaining four studies (16 %) [39–42]. A table including the risk of bias of studies is available and described in Additional file 4: Table S2.

## Synthesis of the results

### Antimalarial drugs for prevention of malaria during pregnancy compared to no use of antimalarial drug and risk of LBW: meta-analysis

**Fig. 2:**
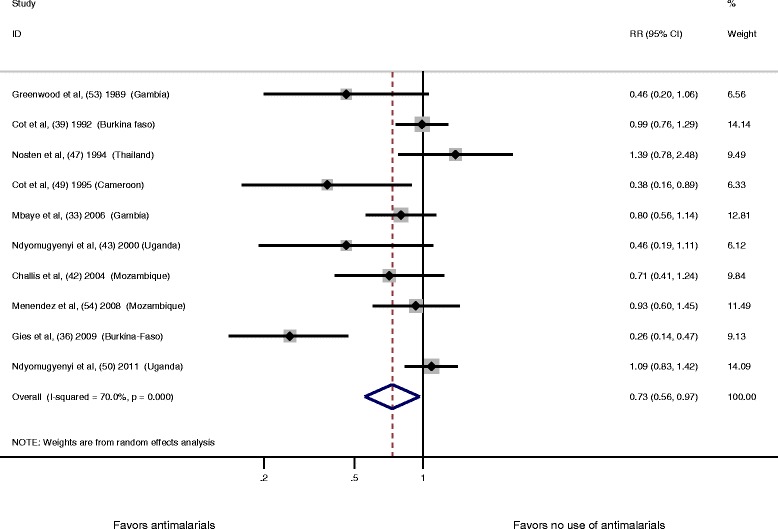
Antimalarial drugs compared to no use of antimalarial drugs and risk of LBW. Each study is displayed as a square and horizontal line, representing the relative risk together with its confidence interval. The area of the square represents the weight that the study contributes to the meta-analysis. The combined relative risk and its confidence interval are represented by the diamond. The *P* value after I^2^ represents chi-square test for heterogeneity. DerSimonian and Laird were used to calculate the random effect model. CI, confidence interval; LBW, low birth weight; RR, relative risk

When all combined antimalarial drugs used for prevention of malaria during pregnancy were compared to no use of antimalarial drug, we detected a significant 27 % reduction in the risk of LBW (RR 0.73, 95 % CI 0.56–0.97; I^2^ = 70 %, *P* <0.01, ten studies) (Fig. 2). As heterogeneity was high (hinging I^2^ statistic = 70 %), we identified an outlier across the ten trials using a Galbraith plot (Additional file 4: Figure S1). The excluded outlying study used a cluster randomized design in which the units of analysis (health centres) were not selected randomly; this may introduce a selection bias. In addition, statistical analysis did not take into account efficiently variability within and between clusters. This may explain why this study was an outlier in our analysis.

After removal of this outlier, heterogeneity between studies decreased from 70 % to 44.1 % (RR 0.86, 95 % CI 0.70–1.05; I^2^ = 44.1 %, *P* = 0.074, nine studies) (Additional file 5: Figure S2). There was a suggestion of a publication bias (Egger’s test, *P* = 0.033; Begg’s test, *P* = 0.032) (Additional file 5: Figure S3), but the trim and fill method indicated no missing studies with a crude estimate similar to the adjusted estimate (Additional file 5: Figure S4 and Table S3). In a sensitivity analysis, we performed a meta-analysis using a fixed effect model, which also showed a significant reduction in the risk of LBW when comparing all the combined antimalarial drugs for prevention of malaria during pregnancy to no use of these drugs (Additional file 5: Figure S5). There was no influence of each individual study on the overall meta-analysis summary estimate after removal of each trial one at a time from the meta-analysis (Additional file 5: Table S4). When we stratified according to the risk of bias, the treatment effect estimate was different in each subgroup of risk of bias with overlapping confidence intervals (test for heterogeneity between subgroups: *P* = 0.075) (Additional file 5: Figure S6), but no statistically significant difference between each risk estimate was seen after using a meta-regression analysis (*P* >0.05) (Additional file 5: Table S5).

### Association between level of drug resistance and gravidity and the efficacy of antimalarial drugs in reducing LBW: meta-analysis

**Fig. 3:**
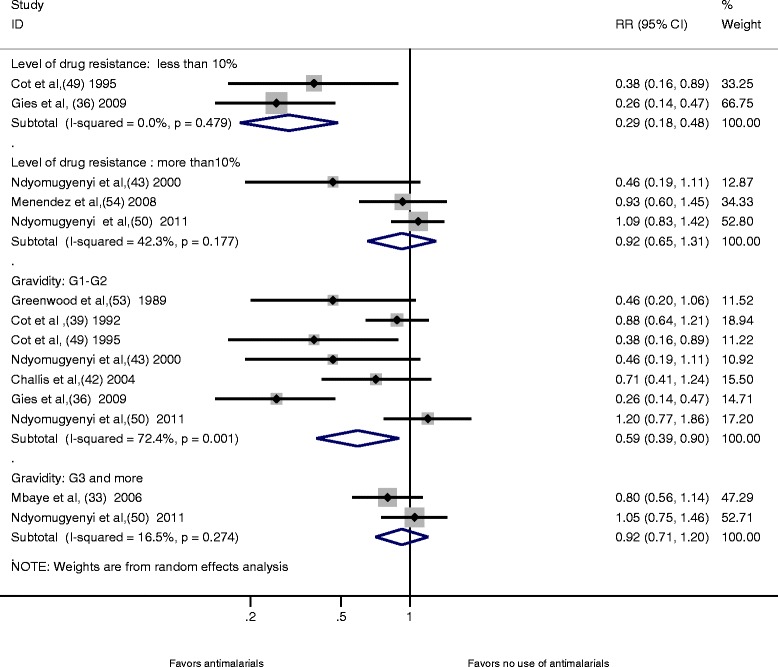
All the combined antimalarial drugs compared to no use of antimalarial drugs and risk of LBW stratified by level of drug resistance and gravidity. Each study is displayed as a square and horizontal line representing the relative risk together with its confidence interval. The area of the square represents the weight that the study contributes to the meta-analysis. The combined relative risk and its confidence interval are represented by the diamond. The *P* value after I^2^ represents chi-square test for heterogeneity. DerSimonian and Laird were used to calculate the random effect model. G1–G2 indicates first and second pregnancy; G3 and more indicates two or more previous pregnancies. Test for subgroup difference between each level of drug resistance (*P* <0.01). Test for subgroup difference between each stratum of gravidity (*P* = 0.102). CI, confidence interval; LBW, low birth weight; RR, relative risk

When all the combined antimalarial drugs used for prevention of malaria during pregnancy were compared to no use of antimalarial drug according to the level of drug resistance based on therapeutic failures at day 14 or 28, we found a significant reduction in the risk of LBW in trials conducted in regions where the level of antimalarial drug resistance was less than 10 % (RR 0.29, 95 % CI 0.18–0.48; I^2^ = 0.0 %, *P* = 0.479, two studies), but not in trials conducted in regions where the level of antimalarial drug resistance was over 10 % (RR 0.92, 95 % CI 0.65–1.31; I^2^ = 42.3 %, *P* = 0.177, three studies) (test for heterogeneity between subgroups: *P* <0.01) (Fig. 3). When sulfadoxine-pyrimethamine was compared to no antimalarial drug use in trials conducted in East Africa, where the level of sulfadoxine-pyrimethamine resistance based on the prevalence of 540E mutation exceeds 50 %, we found no significant reduction of the risk of LBW (RR 0.99, 95 % CI 0.80–1.22; I^2^ = 0.0 %, *P* = 0.376, three studies) (Additional file 5: Figure S7). After stratifying by gravidity, we also noted a significant reduction in the risk of LBW in primigravidae and secundigravidae (RR 0.59, 95 % CI 0.39–0.90; I^2^ = 72.4 %, *P* = 0.01, seven studies), but there was no reduction among higher-order multigravidae (RR 0.92, 95 % CI 0.71–1.20; I^2^ = 16.5 %, *P* = 0.274, three studies); however, confidence intervals overlapped (test for heterogeneity between subgroups: *P* = 0.102) (Fig. 3).

### Three doses or more of the main types of antimalarial drugs compared to two doses of these drugs and risk of LBW: meta-analysis

**Fig. 4:**
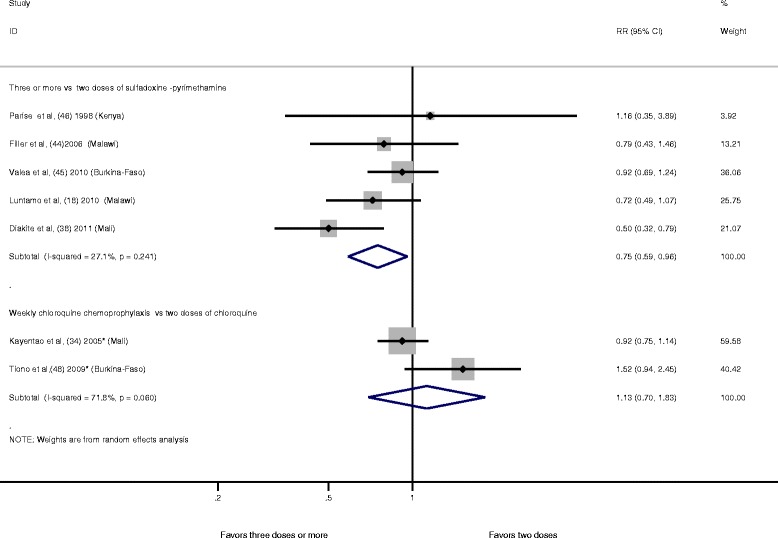
Three doses or more compared to two doses of the main type of antimalarial drug used for prevention of malaria during pregnancy and risk of LBW. Each study is displayed as a square and horizontal line representing the relative risk together with its confidence interval. The area of the square represents the weight that the study contributes to the meta-analysis. The combined relative risk and its confidence interval are represented by the diamond. The *P* value after I^2^ represents chi-square test for heterogeneity. DerSimonian and Laird were used to calculate the random effect model. *Relative risk has been calculated with the Mantel–Haenszel method instead of using the odds ratio (OR) provided in the paper. CI, confidence interval; LBW, low birth weight; RR, relative risk

When three doses or more of sulfadoxine-pyrimethamine were compared to two doses of this drug, we found a significant 25 % reduction in the risk of LBW (RR 0.75, 95 % CI 0.59–0.96; I^2^ = 27.1 %, *P* = 0.241, five studies) (Fig. 4). No evidence of publication bias was detected after inspection of the funnel plot (Egger’s test, *P* = 0.901; Begg’s test, *P* = 1.000) (Additional file 5: Figure S8). In a sensitivity analysis, a significant reduction of LBW was also found when using a fixed effect model (RR 0.77, 95 % CI 0.63–0.93; I^2^ = 27.1 %, *P* = 0.241, five studies) (Additional file 5: Figure S9). There was no influence of each individual study on the overall meta-analysis summary estimate after removal of each trial one at a time from the meta-analysis (Additional file 5: Table S6).

When weekly chloroquine chemoprophylaxis was compared to two doses of chloroquine, no significant reduction in the risk of LBW was found (RR 1.13, 95 % CI 0.70–1.83, two studies) (Fig. 4).

### Sulfadoxine-pyrimethamine compared to antimalarial drug alternatives for malaria prevention during pregnancy and risk of LBW: meta-analysis

**Fig. 5:**
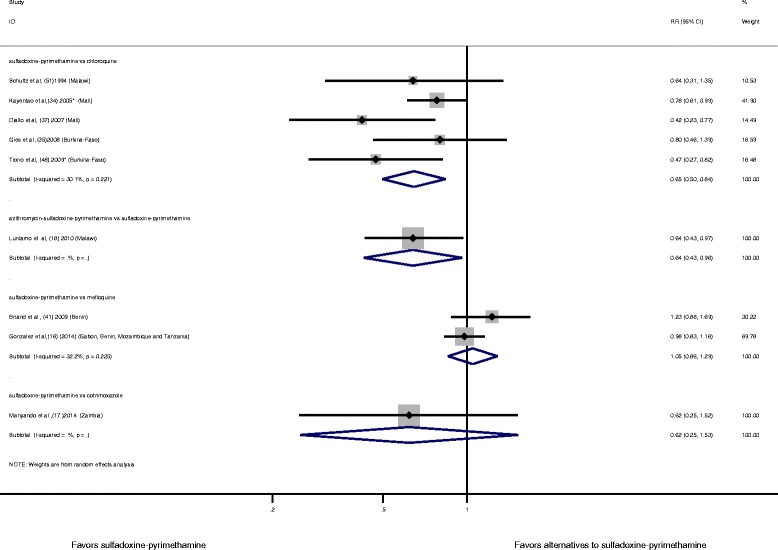
Sulfadoxine-pyrimethamine compared to different antimalarial drug alternatives for malaria prevention during pregnancy and the risk of LBW. Each study is displayed as a square and horizontal line representing the relative risk together with its confidence interval. The area of the square represents the weight that the study contributes to the meta-analysis. The combined relative risk and its confidence interval are represented by the diamond. The *P* value after I^2^ represents chi-square test for heterogeneity. DerSimonian and Laird were used to calculate the random effect model. *Relative risk has been calculated with the Mantel–Haenszel method instead of using the odds ratio (OR) provided in the paper. CI, confidence interval; LBW, low birth weight; RR, relative risk

Compared to chloroquine, sulfadoxine-pyrimethamine was significantly associated with a 40 % reduction in the risk of LBW (RR 0.65, 95 % CI 0.50–0.84; I^2^ = 30.1 %, *P* = 0.221, six studies) (Fig. 5). After visual inspection of the funnel plot, there was no evidence of publication bias using Egger’s test (*P* = 0.389) and Begg’s test (*P* = 0.327) (Additional file 5: Figure S10). In addition, we conducted a sensitivity analysis using a fixed effect model, which showed a reduction of LBW when sulfadoxine-pyrimethamine was compared to chloroquine (RR 0.61, 95 % CI 0.48–0.77) (Additional file 5: Figure S11). When sulfadoxine-pyrimethamine was compared to mefloquine, the risk of LBW was similar between the two groups (RR 1.05, 95 % CI 0.86–1.29; I^2^ = 32.2 %, *P* = 0.225, two studies) (Fig. 5).

There was no association with a reduction in the risk of LBW when sulfadoxine-pyrimethamine was compared to sulfamethoxazole-trimethoprim (RR 0.62, 95 % CI 0.25–1.52, one study) (Fig. 5).

When the combination azithromycin-sulfadoxine-pyrimethamine was compared to sulfadoxine-pyrimethamine alone, we detected a significant reduction in the risk of LBW (RR 0.64, 95 % CI 0.43–0.97, one study) (Fig. 5).

## Discussion

This systematic review and meta-analysis showed that antimalarial drugs used for malaria prevention during pregnancy was associated with a 27 % reduction in the risk of LBW when compared to no use of these drugs. These findings corroborate a previous Cochrane review, which found an association between the use of these drugs and the reduction of the risk of LBW in women in their first and second pregnancy (RR 0.73, 95 % CI 0.61–0.87, eight studies) [6]. Our reviews showed that antimalarial drugs were effective at reducing the risk of LBW in regions where the level of drug resistance was less than 10 %, but not in regions with a level of drug resistance over 10 %. However, our results should be interpreted with caution given the limited number of studies included in each subgroup.

Similar findings were reported recently in a study showing that the effectiveness of sulfadoxine-pyrimethamine in preventing LBW varied with level of resistance to this medication [11]. In contrast, an earlier meta-analysis published in 2007 showed that evaluating sulfadoxine-pyrimethamine resistance on the efficacy of intermittent preventive therapy for malaria control during pregnancy found no association with the level of drug resistance [10]. However, this finding was based on a small number of studies conducted in a region with a narrow range of resistance to sulfadoxine-pyrimethamine (19–26 %), which may explain the lack of association. In addition, we demonstrated that sulfadoxine-pyrimethamine was not associated with a reduction in the risk of LBW in East Africa, where the prevalence of dihydropteroate synthase 540E mutation exceeds 50 %. Moreover, the sextuple mutant parasite has also been recently reported in this region [12]. Indeed, it is known that these mutations may interfere with sulfadoxine-pyrimethamine effectiveness [12–15].

Three RCTs were included in our meta-analysis of East Africa [41, 49, 53]. The first one was conducted in Kabale in Uganda [49], a region where sextuple mutant haplotype (dhps 540E mutation with additional dhps 581G) were prevalent [12]. But, the second and third RCTs were performed in Mozambique where this mutation has not been reported yet [41, 53]. Nonetheless, as it might take an average of 5 years between the collection of information on molecular markers and the date of publication of the results [12], the mutation might be present in Mozambique but not yet detected.

In contrast, another meta-analysis comparing the risk of LBW between women taking three or more doses of sulfadoxine/pyrimethamine to those taking two doses reported a similar risk of LBW in regions with prevalence of dihydropteroate synthase 540E mutation of less or over 50 % [7]. A possible explanation was that an extra dose of sulfadoxine-pyrimethamine compensates for any reduction in efficacy of the two-dose regimen resulting from a progressive decrease of the duration of post-treatment prophylaxis. Nevertheless, this finding should be interpreted with caution. Indeed, it was based on a subgroup analysis with only two studies conducted in regions where the prevalence of dhps 540E mutation was over 50 % and five studies conducted in regions where the prevalence of dhps 540E mutation was less than 50 %. Therefore, a lack of statistical power could not be completely ruled out. However, the lack of association found in that study may also be explained by the fact that the two RCTs were conducted in regions where sextuple mutant parasites were absent.

Of note, East Africa is also a region where the prevalence of HIV infection is high; therefore, our findings highlight the need to address the association between HIV and malaria co-infection and the occurrence of adverse pregnancy outcomes, such as LBW. Our study failed to demonstrate an interaction between gravidity and the effect of antimalarial drugs on the risk of LBW; however, this finding should be interpreted with caution given the low statistical power.

Compared to two doses of sulfadoxine-pyrimethamine, three doses or more was significantly associated with a 25 % reduction in the risk of LBW in this review. This result was similar to a recent meta-analysis, which showed a lower risk of LBW when three doses or more of sulfadoxine-pyrimethamine were compared to two doses [7].

When sulfadoxine-pyrimethamine was compared to mefloquine, the most promising alternative for malaria prevention during pregnancy, the risk of LBW was similar between the two groups. In contrast, when combination azithromycin-sulfadoxine-pyrimethamine was compared to sulfadoxine-pyrimethamine alone, we noted a 36 % reduction in the risk of LBW. However, we could not draw a definite conclusion given the limited data available in the literature. Furthermore, additional medicines for the prevention of malaria in pregnancy are the subject of trials underway through the Malaria in Pregnancy (MiP) Consortium.

To our knowledge, this is the most comprehensive and updated synthesis of all antimalarial drugs used for the prevention of malaria during pregnancy and the risk of LBW. In addition, our study evaluated the association between antimalarial drug resistance (based on treatment failures of antimalarial drugs at day 14 or 28 and on the prevalence of molecular markers of sulfadoxine-pyrimethamine resistance in symptomatic children aged 6–59 months) and gravidity, and the protective effect of antimalarial drugs in reducing LBW. Furthermore, we attempted to explain heterogeneity between the studies and performed sensitivity analyses that showed the robustness of our results.

Nevertheless, this review has some limitations. First, measurement of antimalarial drug resistance was performed on infected children with acute malaria aged 6–59 months, which may not reflect the true resistance in asymptomatic pregnant women as they might still have partial immunity. Given that, it is currently recommended to use asymptomatic pregnant women instead of children under 5 years of age to assess the efficacy of IPT during pregnancy with sulfadoxine-pyrimethamine at different study sites [54]. Therefore, efforts are made to test and incorporate this new approach within national malaria control programs across Africa [55]. However, translating WHO recommendations into national malaria policy remains challenging. A study showed a discrepancy between current WHO recommendations during pregnancy and the national malaria policy in five African countries [56]. Therefore, considering that no alternative for sulfadoxine-pyrimethamine will be available soon, the use of therapeutic failure to sulfadoxine-pyrimethamine in children under 5 years old still brings additional evidence to determine the threshold at which this drug fails to provide any benefit during pregnancy, especially in sites where data on molecular markers of *P. falciparum* resistance to sulfadoxine-pyrimethamine are not yet available, or data on efficacy of IPT with sulfadoxine-pyrimethamine measured among asymptomatic pregnant women in malaria sentinel sites are lacking [11].

Second, data on folic acid use, impregnated bed nets or the pattern of transmission of malaria were not available for each study to assess whether these factors may affect the protective effect of antimalarial drug in reducing LBW.

Third, approximately two-thirds of the studies included in this systematic review had a high risk of bias; however, after stratification of studies according to the risk of bias (high, unclear and low risk of bias) in the main meta-analysis, we found no significant difference between risk estimates of each subgroup using a meta-regression analysis. Although there was a limited number of RCTs in each stratum of risk of bias, a recent simulation study showed that linear regression models require only two subjects per variable for an adequate estimation of regression coefficients, standard errors and confidence intervals [57]. Given that a meta-regression concept is similar to simple linear regression [58], we are confident that our analysis gave an unbiased regression coefficient estimate even though it does not rule out a lack of statistical power. In addition, two out of four (50 %) studies with low risk of bias included in our main meta-analysis were conducted in regions where the prevalence of dihydropteroate synthase 540E exceeds 50 %. This may also explain the variability of the risk estimates between studies stratified according to the risk of bias (clinical heterogeneity).

Fourth, the median dropout rate of studies included in the review was 14.1 %. Furthermore, half of the studies reported using intention-to-treat analysis, but it was in fact an available case analysis because data were analyzed according to the assigned intervention for every participant for whom the outcome was obtained and no missing data were imputed. This may introduce a selection bias and limit the generalizability of this finding. However, when the rate of loss to follow-up and the rate of adverse events and foetal loss were provided in those trials, there was no significant difference between the exposed group and the control group in our main meta-analysis.

Fifth, we did not include unpublished data in our review, which may introduce a reporting bias in meta-analyses. Nevertheless, there was no evidence of publication bias when using a funnel plot or trim and fill method.

Sixth, there was no control for the dose or the method of delivery of mefloquine (IPT or weekly) in the study conducted by Nosten and colleagues in Thailand, which is a source of potential bias. However, when each study was removed one at a time from the main meta-analysis, there was no influence of each individual study on the overall meta-analysis summary estimate.

Seventh, the safety of antimalarial drugs during pregnancy is a concern. Despite the fact that many RCTs have reported antimalarial side effects, very few use appropriate methods of pharmacosurveillance to detect and evaluate safety signals. Therefore, there is a need to implement standardized methods of collecting and reporting adverse events in RCTs, which will help to build a centralized pharmacovigilance database and efficiently identify safety concerns.

Eighth, LBW may be a consequence of small for gestational age (SGA), preterm birth (PTB), or a combination of both. SGAs are not systematically reported in RCTs as an outcome of interest. In our review, only one RCT has reported SGA as one of the outcomes [46]. However, a recent Cochrane systematic review and meta-analysis showed that antimalarial drugs were not associated with PTB [6]. Another meta-analysis also showed no difference in PTB when three doses or more of sulfadoxine-pyrimethamine were compared to two doses of this drug [7]. Therefore, the association found with LBW may mainly reflect an association with fetal growth rather than with PTB. Nonetheless, further RCTs assessing both SGA and PTB are needed.

Ninth, there is a potential for misclassification of the outcome of LBW as the timing of measurement, method of gestational age estimation and details on the scales used for birth weight were not often provided in RCTs included in our review. This may introduce a non-differential outcome misclassification, which may bias estimates toward a null effect. However, this limitation regarding timing of measurement, method of gestational estimation and scales used for birth weight is common for RCTs conducted among pregnant women in malaria-endemic regions [59]. This highlights the need for a standardized method for birth weight measurement and reporting in future studies.

## Conclusions

In summary, prophylactic antimalarial drug use during pregnancy is associated with a reduction in the risk of LBW. There was a dose–response relationship in terms of risk reduction of LBW when three doses or more of sulfadoxine-pyrimethamine were compared to two doses. However, the level of antimalarial drug resistance but not gravidity modified the protective effect of antimalarial drug used for prevention of malaria during pregnancy in reducing the risk of LBW. Sulfadoxine-pyrimethamine may no longer be effective at preventing the risk of LBW in East Africa. To date, there are no suitable alternative drugs to replace sulfadoxine-pyrimethamine for malaria prevention during pregnancy in Africa but additional medicines for the prevention of malaria in pregnancy are the subject of trials underway through the MiP Consortium.

Our study supports the current WHO recommendation for IPT with three doses or more of sulfadoxine-pyrimethamine during pregnancy for malaria prevention. However, there is an urgent need to re-evaluate the efficacy of IPT during pregnancy with sulfadoxine-pyrimethamine, especially in East Africa given the increase of resistance. Also, additional medicines to replace this medication are needed as no suitable alternative drug is available. The continued monitoring of the effectiveness of IPT during pregnancy with sulfadoxine-pyrimethamine in Africa is essential.

## Additional files

Additional file 1:
**PRISMA checklist.** (DOCX 30 kb) Additional file 2:
**Search strategy, PubMed.** (DOCX 12 kb) Additional file 3:
**A summary of included studies.** (DOCX 44 kb) Additional file 4:
**Risk of bias assessment of each RCT.** (DOCX 26 kb) Additional file 5:
**Sensitivity analysis and test for publication bias.** (DOCX 85 kb)

## Abbreviations

CI: Confidence interval
dhfr: Dihydrofolate reductase
dhps: Dihydropteroate synthase
DSMB: Data and safety monitoring board
IPT: Intermittent preventive treatment
IPTi: Intermittent preventive treatment of infants
IQR: Interquartile range
IUGR: Intrauterine growth restriction
LBW: Low birth weight
MDG: Millennium Development Goal
MiP: Malaria in Pregnancy
OR: Odds ratio
PRISMA: Preferred Reporting Items for Systematic Reviews and Meta-Analysis
PTB: Preterm birth
RCT: Randomized controlled trial
RR: Risk ratio
SGA: Small for gestational age
WHO: World Health Organization
