# Supplementary material for: Antimalarial drugs for preventing malaria during pregnancy and the risk of low birth weight: a systematic review and meta-analysis of randomized and quasi-randomized trials
Source: BMC Med. 2015 Aug 14;13:193. doi: 10.1186/s12916-015-0429-x (PMC4537579; doi:10.1186/s12916-015-0429-x)
Supplement: Additional file 2: — Search strategy, PubMed. (DOCX 12 kb) [file 12916_2015_429_MOESM2_ESM.docx]

**Search strategy: PubMed**

("malaria"[MeSH Terms] OR "malaria"[All Fields]) AND ("infant, low birth weight"[MeSH Terms] OR ("infant"[All Fields] AND "low"[All Fields] AND "birth"[All Fields] AND "weight"[All Fields]) OR "low birth weight infant"[All Fields] OR ("low"[All Fields] AND "birth"[All Fields] AND "weight"[All Fields]) OR "low birth weight"[All Fields])
