# Supplementary material for: Antimalarial drugs for preventing malaria during pregnancy and the risk of low birth weight: a systematic review and meta-analysis of randomized and quasi-randomized trials
Source: BMC Med. 2015 Aug 14;13:193. doi: 10.1186/s12916-015-0429-x (PMC4537579; doi:10.1186/s12916-015-0429-x)
Supplement: Additional file 3: — A summary of included studies. (DOCX 44 kb) [file 12916_2015_429_MOESM3_ESM.docx]

| **Authors and publication year** | **Location Study period** | **Study design** | **N total** | **Comparison groups** | **Adjusted for:** | **Risk of LBW** | | **RR** | **95% CI** | **Comments** |
| --- | --- | --- | --- | --- | --- | --- | --- | --- | --- | --- |
|  |  |  |  |  |  | **Exposed group** | **Control group** |  |  |  |
| Greenwood et al, 1989[52] | Gambia  1989 | RCTs | 1049 | Weekly P+DA VS placebo | NA | 8/172(4·6%) | 15/149(10%) | 0.46^a^ | 0.20-1.06 | MT= Mesoendemic *  DR= not reported  DOR, No (%) = 319 /1049(30.4%) |
| Cot et al, 1992[38] | Burkina-Faso  1987-1988 | RCTs | 1464 | Weekly CQ vs no use of CQ | Stratification by gravidity  G1 | 97/595(16·3%)  NA | 91/554(16·4%)  NA | 0·99 ^a^  0.88 ^a^ | 0·76-1·29  1.21-0.64 | MT=hyperendemic **  DR= not reported  DOR, No (%) = 315/1464 (21.5%) |
| Nosten et al, 1994[46] | Thailand  1987-1990 | RCTs | 339 | MQ vs placebo | NA | 24/146(16 %) | 17/144(12 %) | 1·39 ^a^ | 0·78-2·48 | MT= hypoendemic ***  DR= not reported  DOR, No (%) = 28/339 (8.2%) |
| Schultz et al, 1994[50] | Malawi  1992 | RCTs | 357 | IPT SP vs weekly CQ | NA | 12/71(17%) | 10/38 (27 %) | 0·64 ^a^ | 0·31-1·35 | MT= holoendemic ****  DR=CQ =80%  SP= not reported  DOR, No (%) = 61/357 (17%) |

| **Authors and publication year** | **Location Study period** | **Study design** | **N total** | **Comparison groups** | **Adjusted for:** | **Risk of LBW** | | **RR** | **95% CI** | **Comments** |
| --- | --- | --- | --- | --- | --- | --- | --- | --- | --- | --- |
|  |  |  |  |  |  | **Exposed group** | **Control group** |  |  |  |
| Cot et al, 1995[48] | Cameroon  1991-1993 | RCTs | 266  primigravidae | Weekly CQ vs no CQ | NA | 6/57(10·5%) | 18/65(27·7 %) | 0·38 ^a^ | 0·16-0·89 | MT= holoendemic ****  DR= 10%  DOR, No (%) = 57/266 (21.4%) |
| Parise et al, 1998[45] | Kenya 1994-1996 | RCTs | 577 | Monthly SP vs IPT SP | NA | 5/85(5·7%) | 5/99(5·1 %) | 1.16 ^a^ | 0.35-3.89 | MT= holoendemic ****  DR= not reported  DOR, No (%) = 175/577 (30.3%) |
| Ndyomugyenyi et al, 2000[42] | Uganda  1996-1998 | RCTs | 860  primigravidae | Weekly CQ vs placebo | NA | 7/166 (4·2%) | 15/169(8·9 %) | 0.46 ^a^ | 0.19-1.11 | MT= hyperendemic **  DR= 18%  DOR, No (%) = 268/860 (31.1%) |
| Challis et al, 2004[41] | Mozambique  2001-2002 | RCTs | 600  primigravidae | IPT SP vs no IPT SP | NA | 19/200(9·5%) | 27/203(13·3%) | 0·71^b^ | 0·41-1·24 | MT= holoendemic ****  DR= not reported  Pr dhps 540E ≥50%  DOR, No (%) = 309/600 (51.5%) |
| Kayentao et al, 2005[33] | Mali  1998-2001 | RCTs | 1163 primigravidae and secundigravidae | IPT SP vs Weekly CQ  Weekly CQ vs IPT CQ | Adjustedfor risk factor: being primigravidae, having short stature, and female sex of the neonate | 86/354(24·3%)  114/365(31%) | 114/365(31 %)  116/343 (33·8%) | 0·78 ^b^  0·92 ^b^ | 0·61-0·99  0·75-1·14 | MT=hyperendemic **  DR= CQ (15%)  SP (5%)  DOR, No (%) = 53/1163 (4.5%) |

| **Authors and publication year** | **Location Study period** | **Study design** | **N total** | **Comparison groups** | **Adjusted for:** | **Risk of LBW** | | **RR** | **95% CI** | **Comments** |
| --- | --- | --- | --- | --- | --- | --- | --- | --- | --- | --- |
|  |  |  |  |  |  | **Exposed group** | **Control group** |  |  |  |
| Filler et al, 2006[43] | Malawi  2002-2005 | RCTs | 698  primigravidae and secundigravidae | Monthly SP vs IPT SP | NA | 23/216(10·6%) | 29/216(13·4 %) | 0·79 ^b^ | 0·43-1·46 | MT= holoendemic ****  DR= 24%  DOR, No (%) = 143/698 (20.4%) |
| Mbaye et al, 2006[32] | Gambia  2002-2004 | RCTs | 2688 multigravidae | IPT SP vs placebo | NA | 51/931(5·4%) | 63/917(6·8%) | 0·80 ^b^ | 0·56-1·14 | MT= hyperendemic**  DR= not reported  DOR, No (%) = 459/2688 (17%) |
| Diallo et al, 2007[36] | Mali  2003-2004 | RCTs | 301 | IPT SP vs CQ | NA | 13/131(9.9%) | 31/132 (23·4%) | 0·42^a^ | 0·23-0·77 | Not reported  DOR, No (%) = 32/301 (10.6%) |
| Tukur et al, 2007[51] | Nigeria  2002 | RCTs | 500 | IPT SP vs CQ+P | NA | 6/174(3·4 %) | 8/180(4·4 %) | 0.75 ^a^ | 0.26-2.10 | MT= holoendemic ****  DR=not reported  DOR, No (%) = 146/500 (29.2%) |
| Clerk et al, 2008[39] | Ghana  2004-2007 | RCTs | 3643 | IPT SP vs AQ  IPT SP vs AQ SP | Adjusted for gravidity, maternal weight, and place of delivery | 68/286(23·8 %)  105/438(24 %) | 52/270(19·3%)  96/425(22·6%) | 1·20 ^a^  1·06 ^a^ | 0·84-1·72  0·83-1·35 | MT=hyperendemic**  DR=AQ = 9·3 %SP = 10·3%  DOR, No (%) = 142/3643 (3.8%) |

| **Authors and publication year** | **Location Study period** | **Study design** | **N total** | **Comparison groups** | **Adjusted for:** | **Risk of LBW** | | **RR** | **95% CI** | **Comments** |
| --- | --- | --- | --- | --- | --- | --- | --- | --- | --- | --- |
|  |  |  |  |  |  | **Exposed group** | **Control group** |  |  |  |
| Gies et al, 2008[34] | Burkina –Faso  2004-2006 | RCTs | 1844 primigravidae and secundigravidae | IPT SP vs Weekly CQ | Adjustment with for parity, season,  sex of the baby, distance, and bed net ownership | 128/687(18·6%) | 132/593(22·3%) | 0·80 ^a^ | 0·46-1·39 | MT=hyper endemic**  DR= CQ (18%)  SP (8·2%)  DOR, No (%) = 299/1844(16.2%) |
| Menendez et al, 2008[53] | Mozambique  2003-2005 | RCTs | 1030 | IPT+ITN vs Placebo+ITN | Adjusted by gravidity | 38/327(11.6%) | 34/313(10.8) | 0.93^b^ | 0.60-1.45 | MT= holoendemic ***  DR=13%  Pr dhps 540E ≥50%  DOR, No (%) = 16/1030(1.5%) |
| Briand et al, 2009[40] | Benin  2005-2008 | RCTs | 1609 | IPT SP vs IPT MQ | Stratification by gravidity  G1-G2  G3 | 72/730(9•8 %) | 59/735(8 %) | 1•23^b*^ | 0•88-1•69 | MT= holoendemic ****  DR=  MQ= 2.5 %  SP= 50%  DOR, No (%) = 79/1609(4.9%) |

| **Authors and publication year** | **Location Study period** | **Study design** | **N total** | **Comparison groups** | **Adjusted for:** | **Risk of LBW** | | **RR** | **95% CI** | **Comments** |
| --- | --- | --- | --- | --- | --- | --- | --- | --- | --- | --- |
|  |  |  |  |  |  | **Exposed group** | **Control group** |  |  |  |
| Tiono et al, 2009[47] | Burkina -Faso  2008-2009 | RCTs | 648 | IPT SP vs weekly CQ  Weekly CQ vs IPT CQ | Adjusted  Adjusted | 16/141(11·4%)  33/137(23·9%) | 33/137(23·9%)  23/145(15·6%) | 0·47 ^b^  1·52 ^b^ | 0·27-0·82  0.94-2.45 | MT= hyperendemic **  DR=  CQ =46.7%  SP= 12.7%  DOR, No (%) = 223/648(34.4%) |
| Gies et al, 2009[35] | Burkina –Faso  2004-2006 | RCTs | 1544  primigravidae and secundigravidae | IPT SP vs. no use of SP | NA | 104/812 (24·5%) | 19/52(36·5%) | 0·26^a^ | 0·14-0·47 | MT= holoendemic ****  DR= 8.2%  DOR, No (%) = 158/1544(10.1%) |
| Luntamo et al, 2010[18] | Malawi  2003-2006 | RCTs | 1320 | Monthly SP vs IPT SP  AZI SP vs IPT SP | Adjusted for no. of previous  pregnancies and malaria at enrolment | 36/394 (9·1%)  32/406(7·9%) | 52/402(12·9%)  52/402 (12·9%) | 0·72^b^  0·64^b^ | 0·49-1·07  0·43-0·97 | MT= holoendemic ****  DR= not reported  DOR, No (%) = 118/1320(8.9%) |
| Valea et al, 2010[44] | Burkina-Faso  2006-2008 | RCTs | 1296 | Three doses SP vs IPT SP | Adjusted for age category, BMI, malaria infection, malaria season at delivery, supplementation and parity category | 66/515(12·8%) | 73/519(14·1%) | 0·92^b^ | 0·69-1·24 | MT= hyperendemic **  DR= 82%  DOR, No (%) = 262/1296(20.2%) |
|  |  |  |  |  |  | 13/136 (9.6%) | 28/235(11.9%) | 0.84^a^ | 0.45-1.54 |  |
|  |  |  |  |  |  |  |  |  |  |  |

| **Authors and publication year** | **Location Study period** | **Study design** | **N total** | **Comparison groups** | **Adjusted for:** | **Risk of LBW** | | **RR** | **95% CI** | **Comments** |
| --- | --- | --- | --- | --- | --- | --- | --- | --- | --- | --- |
|  |  |  |  |  |  | **Exposed group** | **Control group** |  |  |  |
| Diakite et al, 2011[37] | Mali  2006-2008 | RCTs | 814 | Three doses or more vs two doses of SP | Stratification  by gravidity:  G1-G2 | 25/378(6·6%)  15/151(9·9%) | 48/360(13·3%)  32/151(21·2%) | 0.50^a^  0.46^a^ | 0.32-0.79  0.26-0.82 | MT=hyperendemic **  DR= low  DOR, No (%) = 31/814(3.8%) |
| Ndyomugyenyi et al, 2011[49] | Uganda  1987-1990 | RCTs | 5775 | IPT SP ITN vs placebo ITN | Stratification by  G1-G2  ≥ G3 | 107/1561(6·85 %)  41/609(6·7%)  64/956(6·7%) | 99/1577(6·28 %)  34/606(5·6 %)  65/929(6·9%) | 1·09^b^  1·20^b^  1·05^b^ | 0·84-1·42  0·77-1·86  0·75-1·46 | MT=mesoendemic***  DR= 18.5%  Pr dhps 540E ≥50%  DOR, No (%) = 549/5775(9.5%) |
| Gonzalez et al, 2014[16] | Gabon, Benin, Mozambique and Tanzania  2009-2013 | RCTs | 4749 | IPT SP vs IPT MQ | Adjusted by country | 177/1398 (12•7%) | 360/2778(13%) | 0·98^b*^ | 0·83-1·16 | Not reported  DOR, No (%) = 573/4749(12%) |
|  |  |  |  |  |  | 128/1289 (9.9%) | 221/2146(10.3%) | 0.97^a^ | 0.79-1.26 |  |
| Manyando et al, 2014[17] | Zambia  2009 | RCTs | 280 | IPT SP vs Daily CTX | NA | 12/127 (9·4%) | 7/121(5·8%) | 0·62^b^ | 0·25-1·52 | MT=hypo endemic****  DR =not reported  DOR, No (%) = 30/280(10.7%) |

Table 1: A summary of included studies evaluating the use of antimalarial drugs for preventing malaria during pregnancy and the risk of LBW.

Abbreviations: LBW, low birth weight; RR , risk ratio; CI, confidence interval; MT, malaria transmission; DR, drug resistance; HIV PR, prevalence of human immunodeficiency virus; RCTs, randomized controlled trials; IPT, intermittent preventive treatment ; SP , sulfadoxine –pyrimethamine; CQ, chloroquine; azi, azithromycin ; AQ , amodiaquine; MQ, mefloquine; P+DA , proguanil plus dapsone; G1-G2, first and second pregnancy;G3, third or more than third pregnancy; NA, not available, Pr dhps 540E ≥50%, trials comparing sulfadoxine-pyrimethamine to no use of antimalarial drugs conducted in countries where the prevalence of the prevalence of the dihydropteroate-synthase 540E mutation exceeds 50 %; WHO, world health organization; *mesoendemic: malaria transmission is seasonal under normal rainfall conditions ; ** hyper endemic: malaria transmission is intense but with periods of no transmission during dry season;***hypoendemic: malaria transmission very intermittent ; **** holoendemic: malaria transmission occurs all year long.

^a^ : per protocol analysis; ^b^: intention to treat analysis, ^b*^: modified intention to treat analysis
