# Supplementary material for: Antimalarial drugs for preventing malaria during pregnancy and the risk of low birth weight: a systematic review and meta-analysis of randomized and quasi-randomized trials
Source: BMC Med. 2015 Aug 14;13:193. doi: 10.1186/s12916-015-0429-x (PMC4537579; doi:10.1186/s12916-015-0429-x)
Supplement: Additional file 4: — Risk of bias assessment of each RCT. (DOCX 26 kb) [file 12916_2015_429_MOESM4_ESM.docx]

| Name and year | Random sequence generation | Allocation concealment | Blinding of participants and personnel* | Incomplete outcome data | Selective reporting | Other bias |
| --- | --- | --- | --- | --- | --- | --- |
| Mbaye,  Gambia^32^ | + | + | + | - | + | + |
| Luntamo,  Malawi^18^ | + | + | - | + | + | - |
| Kayentao,  Mali^33^ | ? | - | ? | - | + | ? |
| Gies,  Burkina-Faso^34^ | - | - | + | - | ? | - |
| Gies,  Burkina-Faso^35^ | - | - | + | - | ? | - |
| Diallo,  Mali^36^ | + | - | ? | ? | ? | ? |
| Filler,  Malawi^43^ | ? | ? | + | - | ? | - |
| Parise,  Kenya^45^ | - | - | ? | - | ? | - |
| Menendez,  Mozambique^53^ | + | + | + | + | + | + |
| Gonzalez,  Gabon, Benin, Mozambique and Tanzania^16^ | + | + | - | + | + | + |
| Manyando,  Zambia^17^ | + | + | - | + | + | - |
| Nosten,  Thailand^46^ | ? | ? | + | + | ? | - |

| Name and year | Random sequence generation | Allocation concealment | Blinding of participants and personnel* | Incomplete outcome data | Selective reporting | Other bias |
| --- | --- | --- | --- | --- | --- | --- |
| Tiono,  Burkina-Faso^47^ | + | + | - | - | ? | - |
| Cot,  Cameroon^48^ | - | - | - | - | + | - |
| Ndyomugyenyi,  Uganda^49^ | + | + | + | + | + | + |
| Schultz,  Malawi^50^ | - | - | - | + | + | - |
| Tukur,  Nigeria^51^ | - | - | - | - | + | - |
| Greenwood,  Gambia^52^ | ? | - | - | - | + | - |
| Valea,  Burkina-Faso^44^ | + | + | - | - | + | - |
| Diakite,  Mali^37^ | + | + | - | + | + | + |
| Cot,  Burkina-Faso^38^ | - | - | - | - | ? | - |
| Clerk,  Ghana^39^ | + | + | - | ? | + | + |
| Briand,  Benin^40^ | ? | + | + | ? | + | + |
| Challis,  Mozambique^41^ | ? | ? | + | ? | ? | ? |
| Ndyomugyenyi,  Uganda^42^ | ? | ? | + | ? | ? | ? |

**Table 2. Risk of bias representing the authors’ judgements about each risk of bias item for each included RCT across the domain**.

Note: This table represents risk of bias assessment for each RCTs included in our review.

*This item assessed both performance bias and detection bias.

Reference numbers refer to those in the main manuscript.
