# Supplementary material for: Antimalarial drugs for preventing malaria during pregnancy and the risk of low birth weight: a systematic review and meta-analysis of randomized and quasi-randomized trials
Source: BMC Med. 2015 Aug 14;13:193. doi: 10.1186/s12916-015-0429-x (PMC4537579; doi:10.1186/s12916-015-0429-x)
Supplement: Additional file 5: — Sensitivity analysis and test for publication bias. (DOCX 85 kb) [file 12916_2015_429_MOESM5_ESM.docx]

**Figure S1: Galbraith Plot: All combined antimalarial drugs compared to no use of antimalarial drugs and risk of LBW stratified by design**

Galbraith plot. The log-risk ratio (b) divided by their standard errors of the 14 studies are plotted against the reciprocal of the standard errors (horizontal axis). Solid lines represent the weighted regression line constrained at 0 with a slope equal to the overall log RR of a fixed effects meta-analysis on our data, and its 95% confidence intervals. The position of the studies in the y-axis indicates their contribution to the Q statistic for heterogeneity. The position of the studies on the x-axis indicates the weight of each study in the meta-analysis

**Figure S2: All combined antimalarial drugs compared to no use of antimalarial drugs and risk of LBW after removing outliers identified by Galbreith plot**

**Note: RR, relative risk; CI, confidence interval. Each study is displayed as a square and horizontal line representing the risk relative together with its confidence interval. The area of the square represents the weight that the study contributes to the meta-analysis. The combined relative risk and its confidence interval are represented by the diamond. P-value after the I-squared represent chi square test for heterogeneity. Der Simonian-Laid used to calculate random effect model.**

**Figure S3: Funnel plot: All combined antimalarial drugs as compared to no use of antimalarial drugs and risk of LBW.**

Egger’s test (P-value =0.033) and Begg’s test (P-value =0.032)

**Figure S4: All combined antimalarial drugs compared to no use of antimalarial drugs and risk of LBW (Trim and fill method)**

**Table 3: All combined antimalarial drugs compared to no use of antimalarial drugs and risk of LBW (Trim and fill method)**

| Filled | | | | | | |
| --- | --- | --- | --- | --- | --- | --- |
| Meta-analysis (exponential form) | | | | | | |
|  | | | | | | |
|  | **Pooled** | **95% CI** | | **Asymptotic** | | **No. of** |
| **Method** | Est | Lower | Upper | z_value | p_value | studies |
| **Fixed** | 0.857 | 0.748 | 0.981 | -2.244 | 0.025 | 10 |
| **Random** | 0.730 | 0.552 | 0.964 | -2.214 | 0.027 |  |
| Test for heterogeneity: Q= 30.862on 59degrees of freedom (p= 0.000) | | | | | | |
| Moment-based estimate of between studies variance = 0.126  **Note:** the table showed the pooled estimates of the meta-analysis using fixed and random effect model after adjusting for a potential publication bias.  **Figure S5: All combined antimalarial drugs compared to no use of antimalarial drugs and risk of LBW using a fixed effect model.**  ****  **Note: RR, relative risk; CI, confidence interval. Each study is displayed as a square and horizontal line representing the risk relative together with its confidence interval. The area of the square represents the weight that the study contributes to the meta-analysis. The combined relative risk and its confidence interval are represented by the diamond. P-value after the I-squared represent chi square test for heterogeneity. An inverse of variance weight used to calculate fixed effect model.**  **Table 4 : all combined antimalarial drugs as compared to no use of antimalarial drugs and risk of LBW after removing one study at a time from meta-analysis.**   \| Study omitted \| Estimate \| 95% Confidence Interval \|  \| \| --- \| --- \| --- \| --- \| \| Gies 2009 \| .85569656 \| .69457942 \| 1.0541871 \| \| Mbaye A 2006 \| .70993912 \| .51352268 \| .98148251 \| \| Cot M 1995 \| .76628256 \| .57935971 \| 1.0135137 \| \| Cot, M 1992 \| .68388629 \| .49018076 \| .95413876 \| \| Greenwood 1989 \| .75447261 \| .56634521 \| 1.0050919 \| \| Challis, K 2004 \| .72717935 \| .53610134 \| .98636168 \| \| Ndyomugyenyi R 2011 \| .67813522 \| .49357352 \| .93170995 \| \| Ndyomugyenyi 2000 \| .7525664 \| .56487077 \| 1.0026296 \| \| Menendez 2008 \| .70025367 \| .51152313 \| .95861787 \| \| Nosten, F. 1994 \| .68244493 \| .50951153 \| .91407371 \| \| Combined \| .72966743 \| .5520308 \| .9644653 \|   **Figure S6: All combined antimalarial drugs for malaria prevention during pregnancy compared to no use of antimalarial drug stratified by the risk of bias of studies included in the meta-analysis.**  ****  **Note: RR, relative risk; CI, confidence interval. Each study is displayed as a square and horizontal line representing the risk relative together with its confidence interval. The area of the square represents the weight that the study contributes to the meta-analysis. The combined relative risk and its confidence interval are represented by the diamond. P-value after the I-squared represent chi square test for heterogeneity. Der Simonian-Laid used to calculate random effect model. Test for subgroup difference between each level of risk of bias (P- value=0.075)**  **Table 5 : meta-regression analysis comparing the treatment effect estimates of antimalarial drugs on the risk of LBW stratified by Cochrane risk of bias domain**   \| logRR \| Coef. \| Std. Err. \| t \| P>t \| 95% confidence interval \|  \| \| --- \| --- \| --- \| --- \| --- \| --- \| --- \| \|  \|  \|  \|  \|  \|  \|  \| \| r1 \| -.4433096 \| .4144449 \| -1.07 \| 0.320 \| -1.423316 \| .5366968 \| \| r3 \| -.5341318 \| .541839 \| -0.99 \| 0.357 \| -1.815377 \| .7471138 \| \| _cons \| .0122234 \| .3495389 \| 0.03 \| 0.973 \| -.8143048 \| .8387516 \| \|  \|  \|  \|  \|  \|  \|  \| | | | | | | |

**r2**: trials with low risk of bias (reference).

**r1**: trials with high risk of bias.

**r3**: trials with unclear risk of bias

**Figure S7: Sulfadoxine-pyrimethamine as compared to no use of antimalarial drugs and the risk of LBW in East Africa**

**Note: RR, relative risk; CI, confidence interval. Each study is displayed as a square and horizontal line representing the risk relative together with its confidence interval. The area of the square represents the weight that the study contributes to the meta-analysis. The combined relative risk and its confidence interval are represented by the diamond. P-value after the I-squared represent chi square test for heterogeneity. Der Simonian-Laid used to calculate random effect model.**

Figure S8: **Funnel plot: three doses or more compared to two doses of sulfadoxine-pyrimethamine and risk of LBW.**

Egger’ stest (P-value = 0.901) and Begg’s test (P-value=1.000)

**Figure S9: three doses or more compared to two doses of sulfadoxine-pyrimethamine and risk of LBW using a fixed effect model.**

**Note: RR, relative risk; CI, confidence interval. Each study is displayed as a square and horizontal line representing the risk relative together with its confidence interval. The area of the square represents the weight that the study contributes to the meta-analysis. The combined relative risk and its confidence interval are represented by the diamond. P-value after the I-squared represent chi square test for heterogeneity. An inverse of variance weight used to calculate fixed effect model.**

**Table 6: three doses or more compared to two doses of sulfadoxine-pyrimethamine and risk of LBW after removing one study at a time from meta-analysis.**

|  |
| --- |
| Study omitted Estimate [95% Conf. Interval] |
|  |
| Diakite et al, (38) 2011 (Mali) 0.84388584 0.67819571 1.0500557 |
| Valea et al, (45) 2010 (Burkina-Faso) 0.66408503 0.51064128 0.86363751 |
| Luntamo et al, (18) 2010 (Malawi) 0.75625843 0.53440881 1.0702046 |
| Filler et al, (44)2006 (Malawi) 0.74028546 0 .54439151 1.0066699 |
| Parise et al, (46) 1998 (Kenya) 0 .7346282 0.56228274 0.95979935 |
|  |
| Combined 0.75187698 0 .58922023 0.95943583 |
|  |

**Figure S10: Funnel Plot; Sulfadoxine-pyrimethamine compared to chloroquine and risk of LBW**

.

Egger’s test (P-value = 0.211) and Begg’s test (P-value=0.221)

**Figure S11: Sulfadoxine-pyrimethamine compared to chloroquine and risk of LBW using a fixed effect model.**

**Note: RR, relative risk; CI, confidence interval. Each study is displayed as a square and horizontal line representing the risk relative together with its confidence interval. The area of the square represents the weight that the study contributes to the meta-analysis. The combined relative risk and its confidence interval are represented by the diamond. P-value after the I-squared represent chi square test for heterogeneity. An inverse of variance weight used to calculate fixed effect model. .* Risk relative has been calculated with Mantel-Haenszel method instead of using Odd ratio (OR) provided in the paper**
